# Home-Based Monitoring of Sleep and Bedroom Temperature in Older Adults

**DOI:** 10.1093/geroni/igaf122.3285

**Published:** 2025-12-31

**Authors:** Pei-An Lee, Peyton Berning, Wanting Yu, Brad Manor, Lewis Lipsitz, Amir Baniassadi

**Affiliations:** Hebrew SeniorLife & Harvard Medical School, Boston, Massachusetts, United States; Hebrew SeniorLife, Boston, Massachusetts, United States; Hebrew SeniorLife, Boston, Massachusetts, United States; Hebrew SeniorLife/Harvard Medical School, Boston, Massachusetts, United States; Hebrew Senior Life, Boston, Massachusetts, United States; Hebrew SeniorLife, Boston, Massachusetts, United States

## Abstract

More than half of older adults complain about sleep problems, while the underlying reason remains undiagnosed in a substantial portion of them. Observational studies conducted in homes have suggested associations between ambient temperature—varying naturally with weather and seasons—and sleep outcomes. Further, a causal link between ambient temperature and sleep has been established within laboratory settings through artificial temperature manipulations. Yet, with many behavioral and environmental confounders, it is not clear whether and to what extent the causal effect of temperature on sleep translates into older adults’ own homes. Fortunately, wearable sleep monitors and cloud-connected thermostats now enable us to remotely monitor sleep while varying bedroom temperature. We piloted this approach in cognitively intact, independent-living older adults aged ≥65 years. Thirteen participants (mean age: 82.6±5 years; 7 females) took part in the study. Over 538 analyzed nights, bedroom temperatures averaged 70.5±2.5 °F (range: 65–76 °F), and overall sleep efficiency (the ratio of time spent asleep to time spent in bed) averaged 89.8±9.6%. Preliminary individual-level analyses revealed a within-person relationship between bedroom temperature and sleep efficiency in 4 of the 11 participants with at least 10 nights of data. These findings demonstrate, for the first time in at-home settings, that older adults may exhibit individual-level sensitivities in sleep efficiency related to bedroom temperature, paving the way for future research aiming to better understand the extent to which sleep in older adults is influenced by environmental factors.

